# Catalytic Pyrolysis of Cellulose Biomass to Aromatic Hydrocarbons Using Modified HZSM-5 Zeolite

**DOI:** 10.3390/nano15100751

**Published:** 2025-05-16

**Authors:** Jian Li, Laizhi Sun, Derun Hua, Xinning Lu, Dandan Yang, Zhiying Wu

**Affiliations:** 1 School of Chemistry and Chemical Engineering, Gannan Normal University, Ganzhou 341000, China; huaderun@gnnu.edu.cn (D.H.); luxinning@gnnu.edu.cn (X.L.); 13676388052@163.com (D.Y.); wuzhiying110@icloud.com (Z.W.); 2 Key Laboratory of Jiangxi University for Functional Materials Chemistry, Ganzhou 341000, China; 3 Shandong Province Key Laboratory of Biomass Gasification Technology, Energy Institute, Qilu University of Technology (Shandong Academy of Science), Jinan 250013, China; sunlz@sderi.cn

**Keywords:** catalytic pyrolysis, biomass, aromatic hydrocarbons, modified HZSM-5

## Abstract

Gallium-modified Zeolite Socony Mobil-5 (ZSM-5) zeolites were synthesized using wetness impregnation and hydrothermal synthesis methods. The structural and acidic properties of the zeolites were characterized through an analytical instrument, which demonstrated that Gallium-modified HZSM-5 zeolites retain the Mobil five instructure (MFI) framework structure, but exhibit a reduction in Brønsted acid sites and a decrease in micropore size. The catalytic performance of these zeolites in the pyrolysis of cellulose biomass and polyethylene was tested. Compared with HZSM-5, Ga-modified HZSM-5 zeolites considerably increased monoaromatic yields while reducing alkanes production. In particular, gallium-impregnated HZSM-5 increased the monoaromatic yield from 37.6% for ZSM-5 to 43.2%, while hydrothermal synthesized Ga-HMFI reduced polyaromatic and alkane yields from 6.6% and 24.6% for HZSM-5 to 2.9% and 11.4%, respectively. These results indicated that Ga-modified HZSM-5 zeolites can improve the synergy between cellulose-derived oxygenates and polyethylene-derived olefins, enhancing the yield of petrochemical hydrocarbons compared to that predicted by theoretical calculations.

## 1. Introduction

As crude oil reserves worldwide continue to deplete rapidly, biomass has attracted considerable research attention as a sustainable energy source. Lignocellulosic biomass, a renewable and carbon-neutral feedstock, holds significant potential as a substitute for conventional petroleum and offers considerable economic value [1,2,3]. Catalytic pyrolysis has emerged as a promising technology for directly converting lignocellulosic biomass into high-quality hydrocarbons, including aromatics and olefins, which serve as valuable precursors for fine chemicals such as organic solvents and polymeric materials [4,5,6]. Consequently, catalytic pyrolysis represents an efficient approach for utilizing lignocellulosic biomass in the production of bio-based hydrocarbons.

Despite its potential, the conversion efficiency of catalytic pyrolysis remains a key challenge. Among the various catalysts investigated, HZSM-5 molecular sieves have been widely applied in petroleum refining for synthesizing liquid fuels and have demonstrated high aromatic yields in the catalytic pyrolysis of biomass [7,8]. However, the carbon atom yield of aromatics and olefins remains limited to approximately 25% and 8%, respectively [6,9,10]. Enhancing the physicochemical properties of HZSM-5 zeolite is therefore essential to suppress the formation of polycyclic aromatics and alkanes, thereby improving the selectivity for petroleum hydrocarbons [5,11].

The low yield of petroleum hydrocarbons in biomass catalytic pyrolysis is primarily attributed to the hydrogen-deficient (~6 wt.%) and oxygen-rich (~40 wt.%) nature of biomass [12,13]. Additionally, significant amounts of solid residues are formed during biomass pyrolysis and the catalytic conversion of oxygenated compounds within the microporous channels of HZSM-5 zeolite [14,15]. The primary pyrolysis products (small oxygen-containing compounds) undergo decarbonylation and decarboxylation reactions at the catalytic sites of the zeolite, leading to the formation of CO and CO_2_. These factors restrict the efficiency of catalytic pyrolysis in producing petrochemical hydrocarbons such as aromatics and olefins.

To address these limitations, recent studies [15,16,17] have explored catalytic co-pyrolysis of waste plastics with biomass model compounds, particularly cellulose, as a synergistic strategy for resource utilization. Polyolefins, a major component of solid organic waste, are characterized by high hydrogen content (~15 wt.%) and negligible oxygen content. The integration of plastics such as polyethylene with cellulose not only increases the hydrogen availability in the reactant mixture but also facilitates the valorization of organic waste. Previous studies [18,19] have demonstrated that co-feeding cellulose with polyethylene significantly enhances the selectivity toward petrochemical hydrocarbons while reducing alkane and solid residue formation.

To further optimize the catalytic efficiency of HZSM-5 zeolite in the pyrolysis of cellulose and polyethylene, various chemical modifications have been explored. Notably, gallium (Ga) modification facilitates the deoxygenation of oxygenated intermediates derived from cellulose pyrolysis [3,20] and enhances aromatic yields during the co-feeding of cellulose and polyethylene [21]. Building on these findings, this study hypothesizes that Ga modification can regulate the physicochemical properties of HZSM-5, thereby improving its shape-selective catalytic performance in catalytic pyrolysis.

To validate this hypothesis, Ga-modified HZSM-5 zeolites were synthesized using incipient wetness impregnation and hydrothermal methods. The structural and textural properties of the zeolites were characterized using X-ray diffraction (XRD), X-ray fluorescence (XRF), Ar adsorption–desorption analysis, and in situ Fourier-transform infrared (FT-IR) spectroscopy with pyridine desorption. Catalytic performance was evaluated using a microreactor system. In addition, the reaction mechanism underlying the co-pyrolysis of cellulose biomass and polyethylene was investigated by varying the polyethylene content in the reactant mixture.

## 2. Materials and Method

### 2.1. Materials

Tetrapropylammonium hydroxide (TPAOH), aluminum nitrate, gallium nitrate, silica gel, sodium hydroxide, and ammonium nitrate were obtained from Aladdin Corporation (Shanghai, China). Cellulose and polyethylene were purchased from Sigma-Aldrich Corporation (St. Louise, MO, USA). HZSM-5 zeolite catalyst (*n*(SiO_2_)/*n*(Al_2_O_3_) = 25) was obtained from Sinopec Catalysts Corporation (Shanghai, China).

### 2.2. Zeolites Preparation

Ga/HZSM-5 zeolite was synthesized via impregnation with a gallium nitrate solution of 0.1 mol/L concentration. During the preparation process, 5 g HZSM-5 zeolite was impregnated with 13.5 mL gallium nitrate solution and stirred at 50 °C until the deionized water evaporated completely. The resulting solid was dried at 110 °C in an oven for 12 h and subsequently calcined at 560 °C in a furnace for 5 h.

Ga-HMFI zeolite was synthesized through previously reported methods [22,23]. The precursor solution was prepared using Tetrapropylammonium hydroxide (TPAOH), aluminum nitrate, gallium nitrate, silica gel, sodium hydroxide, and deionized water, with an initial composition of 50SiO_2_: 1Ga_2_O_3_: 1Al_2_O_3_: 2Na_2_O: 10TPAOH: 3000H_2_O. The resulting gel was heated at 190 °C in an autoclave (Songling Chemical Equipment, Yantai, China) for 60 h, followed by filtration and washing with deionized water. The solid products were dried at 110 °C for 12 h and calcined at 550 °C for 4 h. Finally, the Ga-HMFI zeolite was converted to H-type form through three successive ion-exchange steps using an aqueous ammonium nitrate solution, followed by filtration, drying in an oven at 110 °C, and calcination in a furnace at 550 °C.

### 2.3. Zeolites Characterization

The crystalline structure of the zeolites was analyzed using a Bruker D8 Advanced powder X-ray diffractometer (Bruker Corporation, Bremen, Germany) with CuKα radiation at 40 kV and 40 mA. Scanning was performed over a 2θ range of 5° to 50°. The elemental composition of the zeolites was determined using an XRF spectrometer (XRF-1800, Shimadzu Corporation, Kyoto, Japan). Textural properties were characterized through liquid argon adsorption at −186 °C using an ASAP2000 instrument (Micromeritics Corporation, Norcross, GA, USA). Surface areas and micropore volumes were calculated through the BET and *t*-plot methods, respectively. The acidity of the zeolites was evaluated through pyridine Fourier transform infrared spectrometer using a Nicolet 5700 instrument (Thermo Fisher Corporation, Waltham, MA, USA). Catalysts wafers (13 mm diameter, 20 mg) were prepared and activated in an IR cell at 400 °C for 2 h under vacuum. Background spectra of the catalysts were recorded before pyridine adsorption. Pyridine was adsorbed for 20 min, followed by desorption at 200 °C. Peaks observed at 1450 cm^−1^ and 1540 cm^−1^ were assigned to Lewis acid sites and Brønsted acid sites, respectively.

### 2.4. Catalytic Pyrolysis

The catalytic pyrolysis of HZSM-5, Ga/HZSM-5, and Ga-HMFI zeolites was conducted using a microreactor (CDS Analytical Corporation, CDS 5200, Oxford, PA, USA). For sample preparation, the zeolite was blended with the reactants (cellulose, polyethylene, or their mixtures) in a 15:1 ratio. High zeolite-to-reactants ratios (e.g., 15–20) were used to ensure complete conversion due to the limited capacity of the CDS pyroprobe [10,18]. Approximately 3.8–4.5 mg of the sample was placed in a pyroprobe, heated to 550 °C, and held at this temperature for 1 min under a helium flow. The resulting pyrolysis products were transported by the helium carrier gas to a gas chromatography instrument (Agilent Technologies Corporation 7890A GC, Santa Clara, CA, USA) equipped with a mass spectrometry (MS, 5975C), a flame ionization detector (FID), and a thermal conductivity detector (TCD). The final condensable and non-condensable products were separated using an HP-5MS column and an HP-Plot/Q column, respectively. The carbon content of the solid residue was determined using an elemental analyzer (Exeter Analytical Corporation, CE-440, North Chelmsford, MA, USA). Each pyrolysis condition was tested in triplicate. The table of product yield presents the total carbon yields and associated standard deviations.

### 2.5. Theoretical Yields of the Major Products

To investigate the interactions between cellulose and polyethylene during catalytic pyrolysis, theoretical yields were calculated for the co-pyrolysis of cellulose with polyethylene (Equation (1)) [24,25]. In this equation, *a* and *b* represent the mass ratios of cellulose biomass and polyethylene in the mixing reactant, respectively. *Y*_cellulose_ and *Y*_polyethylene_ denote the average product yields from the individual catalytic pyrolysis of cellulose biomass and polyethylene. *C*_cellulose_, *C*_polyethylene_, and *C*_mixture_ correspond to the carbon atom content in cellulose, polyethylene, and the mixture, respectively.

(1) $$Y_{theoretical}\%=\frac{aY_{\mathrm{cellulose}}C_{\mathrm{cellulose}}\%+bY_{\mathrm{polyethylene}}C_{\mathrm{polyethylene}}\%}{(a+b)C_{\mathrm{mixture}}\%}\times 100\%$$

## 3. Results

### 3.1. Zeolite Characterization

**Figure 1:**
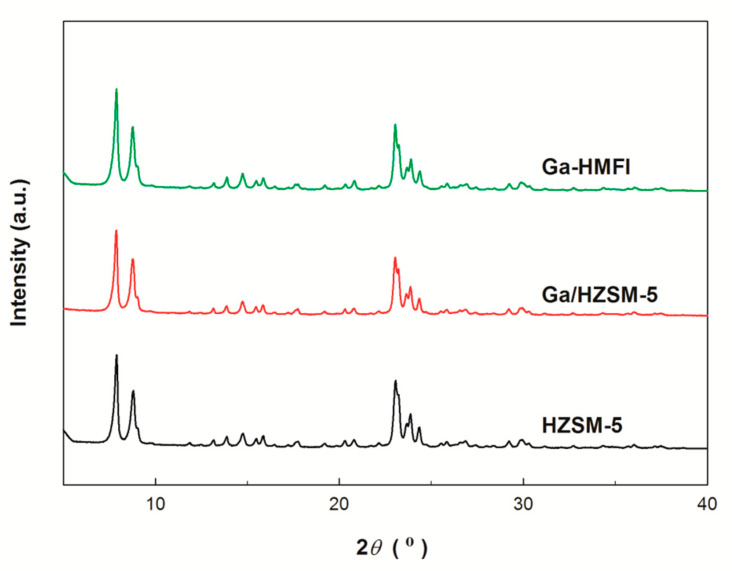
XRD patterns of HZSM-5 and Ga-modified zeolites.

As shown in Figure 1, Ga/HZSM-5 zeolite exhibits the same MFI-type structure as HZSM-5. Notably, no diffraction peaks corresponding to bulk Ga_2_O_3_ crystallites (typically observed at ~32° and ~35°) are detected, indicating the homogeneous dispersion of non-framework gallium oxide on the external surface of the zeolite [20,26]. Furthermore, the hydrothermally synthesized Ga-HMFI zeolite also exhibits characteristic MFI-type diffraction peaks, with no detectable impurity phases. These results confirm that the Ga-HMFI zeolite framework consists of a pure MFI-type structure, in which silicon, gallium, and aluminum atoms are coordinated through oxygen bridges.

The elemental composition and framework characteristics of the zeolites are summarized in Table 1. The Ga/HZSM-5 zeolite contains 4.82% gallium oxides, which is impregnated onto the HZSM-5 framework. Compared with conventional HZSM-5, the micropore surface area and micropore volume of Ga/HZSM-5 decrease to 336.3 m^2^/g and 0.130 cm^3^/g, respectively. This reduction in microporosity is primarily attributed to the decomposition of gallium nitrate during calcination, leading to the formation of gallium oxides that are dispersed on the crystallite surfaces and micropore openings [25]. Additionally, the hydrothermally synthesized Ga-HMFI zeolite exhibits a SiO_2_/(Al_2_O_3_ + Ga_2_O_3_) molar ratio of 28.7. Previous studies have demonstrated that the removal of organic templates during calcination induces partial extraction of gallium from the framework, leading to the formation of non-framework gallium oxides that deposit within micropores and on the external surface [14,20]. Consequently, Ga-HMFI zeolite contains both non-framework gallium oxide species and gallium incorporated within the framework.

**Figure 2:**
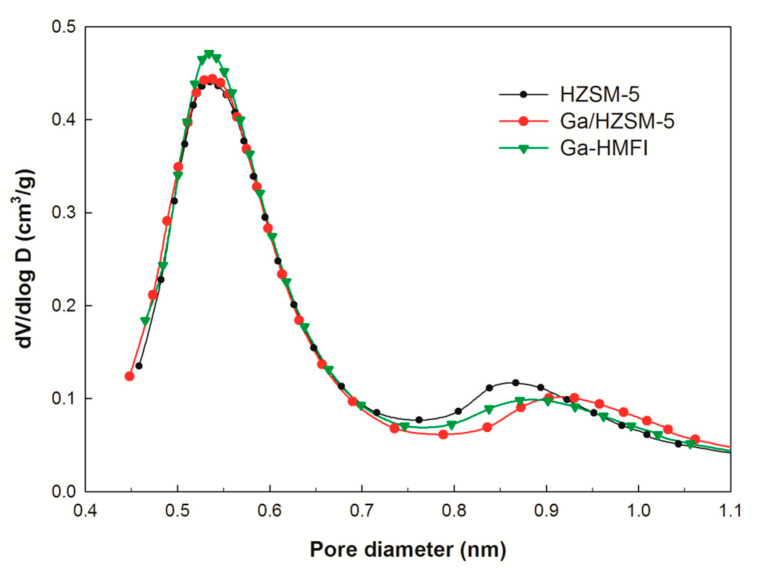
Micropore distribution of HZSM-5 and Ga-modified zeolites.

The pore size distribution of the zeolites, as determined by Ar adsorption–desorption analysis, is presented in Figure 2. Pores of approximately 0.55 nm correspond to the straight and sinusoidal channels of the MFI structure, while pores around 0.80–0.9 nm are attributed to the intersections of these channels [8]. The intersection density of Ga/HZSM-5 is lower than that of conventional HZSM-5, suggesting that the impregnated gallium oxides preferentially deposit at these intersections rather than within the micropore channels [15,27]. In contrast, Ga-HMFI zeolite exhibits a slightly higher distribution of ~0.55 nm pores compared with both HZSM-5 and Ga/HZSM-5. This increase is primarily attributed to the partial occupation of micropores by non-framework gallium oxides [20].

**Figure 3:**
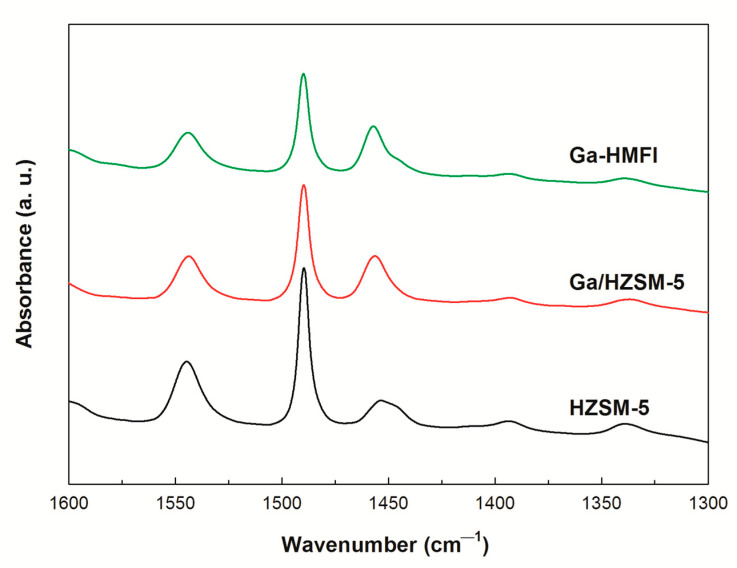
Py-IR spectra of HZSM-5 and Ga-modified zeolites.

Acidity measurements obtained from pyridine-IR spectra are shown in Figure 3. Three distinct desorption peaks are observed at 1450 cm^−1^, 1540 cm^−1^, and 1490 cm^−1^, corresponding to Lewis acid sites (L), Brønsted acid sites (B), and a combined contribution from both B and L acid sites, respectively [28]. In Ga/HZSM-5 zeolite, the partial occupation of external acid sites by deposited gallium oxides results in a decrease in B acid site density compared to HZSM-5. Additionally, Ga-HMFI zeolite exhibits a lower B acid site density than HZSM-5, as bridged gallium hydroxyl groups exhibit greater covalency than bridged silanol groups [3,26]. In contrast, the increase in L acid site density observed in both Ga/HZSM-5 and Ga-HMFI zeolites is primarily attributed to the presence of non-framework gallium oxides [26,27].

The physicochemical characterization results confirm that both gallium-impregnated Ga/HZSM-5 and hydrothermally synthesized Ga-HMFI zeolites retain an intact MFI-type framework, with no evidence of bulk gallium oxides formation. Notably, Ga modification significantly affects the porosity and acidity of the zeolites, which in turn influences their shape selectivity in the catalytic pyrolysis process of cellulose and polyethylene. These effects are further discussed in the following section.

### 3.2. Catalytic Pyrolysis Tests

#### 3.2.1. Catalytic Pyrolysis of Individual Cellulose and Polyethylene

Table 2 summarizes the major products obtained from the individual catalytic pyrolysis of cellulose or polyethylene over the examined zeolites. These products can be categorized into aromatics (comprising monocyclic aromatic hydrocarbons and polycyclic aromatic hydrocarbons), lower-carbon olefins and alkanes, CO_x_ (CO and CO_2_), and solid residues (char and coke). Notably, oxygenated compounds derived from cellulose pyrolysis were not detected, indicating their complete deoxygenation by the acid sites of the zeolites. The final product distribution is strongly influenced by the shape-selectivity of the catalysts.

As shown in Table 2, the monoaromatic yield from catalytic pyrolysis of cellulose increased from 19.2% with HZSM-5 to 25.9% with Ga/HZSM-5. This enhancement is primarily attributed to the presence of gallium oxide species, which promote the dehydrogenation of oxygenated intermediates, facilitating aromatization. In contrast, Ga-HMFI zeolite produced only 4.0% polyaromatics, significantly lower than the 9.7% and 10.0% observed with HZSM-5 and Ga/HZSM-5, respectively. Compared to the other zeolites, Ga-HMFI exhibited a higher micropore distribution (~0.55 nm), as determined by Ar adsorption–desorption analysis (Figure 2). The framework of Ga-HMFI sterically hinders the growth of bulky polyaromatic hydrocarbons by inhibiting their formation in the narrowed micropores or preventing them from diffusing out of these micropores [23], thereby restricting their production.

Previous studies [3,29] have demonstrated that cellulose thermal decomposition generates small oxygenates that diffuse into the micropores of zeolites, where they undergo deoxygenation, cracking, polymerization, and aromatization catalyzed by the solid acid sites [18,30]. The acidity of the zeolites plays a crucial role in determining product selectivity. Although Ga-modified zeolites exhibit lower Brønsted acidity than HZSM-5, their shape selectivity favors the formation of petroleum hydrocarbons. This result suggests that non-framework gallium oxides significantly enhance the catalytic conversion of olefinic intermediates into high-value hydrocarbons [14].

The catalytic pyrolysis of polyethylene over these zeolites yielded similar products, including aromatics, olefins, alkanes, and solid residues (Table 2). It has been reported [18,19] that polyethylene pyrolysis primarily generates a range of branched hydrocarbons, which undergo further cracking and reforming on the external acid sites of the zeolite. The resulting olefins diffuse into the zeolite micropores, where they are converted into final products through oligomerization, cyclization, and aromatization.

As shown in Table 2, catalytic pyrolysis of polyethylene over conventional HZSM-5 produced a monoaromatic yield of only 24.2%, whereas Ga/HZSM-5 and Ga-HMFI zeolites exhibited significantly higher yields of 34.0% and 35.2%, respectively. Additionally, the olefin yield increased from 14.7% with Ga/HZSM-5 to 31.6% with Ga-HMFI, while the alkane yield decreased from 42.4% to 25.2%. These results indicate that gallium modification has a profound effect on product distribution during polyethylene pyrolysis.

Compared with HZSM-5, Ga/HZSM-5 and Ga-HMFI reduced remarkably alkane yields while enhancing monoaromatic and olefin production. These trends can be attributed to the role of non-framework gallium oxides in promoting alkane dehydrogenation and subsequent aromatization [20,31], which drive the conversion of olefins into aromatics. Thus, Ga-modified zeolites effectively enhance the yields of petroleum hydrocarbons.

As expected, cellulose pyrolysis generated considerably higher solid residue yields (30.0–36.3%) compared to polyethylene (2.6–7.0%) (Table 2). This disparity is primarily due to the lower hydrogen content of cellulose (~6.2 wt.%) [16,18], which leads to substantial coke formation during catalytic upgrading. Previous studies [10,16] have demonstrated that co-feeding cellulose with polyethylene induces a synergistic effect that reduces coke formation while increasing the yield of petroleum hydrocarbons. To optimize this synergy, the catalytic performance of HZSM-5, Ga/HZSM-5, and Ga-HMFI zeolites was further evaluated in the catalytic pyrolysis of cellulose–polyethylene mixtures.

#### 3.2.2. Catalytic Pyrolysis of Cellulose Co-Feeding with Polyethylene

**Figure 4:**
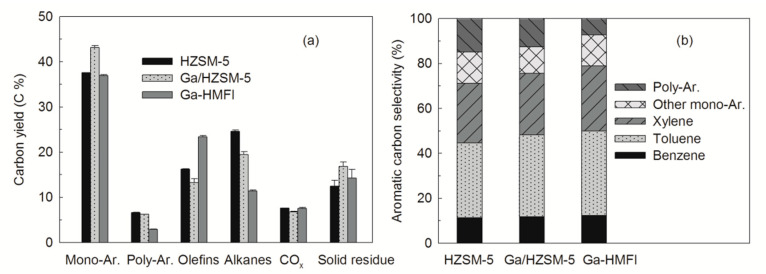
Major products from catalytic pyrolysis of cellulose and polyethylene (mass ratio of 1:1) over HZSM-5 and Ga-modified zeolites ((**a**) carbon yield, and (**b**) aromatic hydrocarbons selectivity). (Mono-Ar.: Monoaromatics; Poly-Ar.: Polyaromatics).

As shown in Figure 4, catalytic pyrolysis of cellulose biomass and polyethylene (mass ratio of 1:1) significantly influenced the distribution of final products. The monoaromatic yield increased from 37.5% with conventional HZSM-5 zeolite to 43.2% with Ga/HZSM-5 zeolite (Figure 4a), while the alkane yield decreased from 24.6% to 19.4%. These trends indicate that the deposition of gallium oxides on Ga/HZSM-5 zeolite not only inhibits the diffusion of polyaromatics but also facilitates alkane dehydrogenation.

Although Ga-HMFI zeolite yielded 37.0% monoaromatics, similar to HZSM-5, it significantly reduced polyaromatic and alkane yields to 2.9% and 11.4%, respectively. Previous studies [16,25] have shown that conventional HZSM-5 zeolite is ineffective at dehydrogenation alkanes, resulting in a considerable alkane yield (24.6%) in the catalytic pyrolysis of cellulose with polyethylene. In contrast, the incorporation of gallium into the Ga-HMFI zeolite structure significantly enhances catalytic dehydrogenation performance, facilitating the dehydrogenation of alkanes to olefins.

Catalytic pyrolysis of the mixtures over these zeolites generated a range of aromatic hydrocarbons, typically classified into five groups: benzene, toluene, xylene, other monoaromatics, and polyaromatics (Figure 4b). Benzene, toluene, and xylene (BTX) are key petrochemical products used in the synthesis of various organic chemicals, including solvents, pharmaceuticals, plastics, and fuel additives [24,32]. The BTX selectivity over HZSM-5 was 71.1%, while Ga/HZSM-5 and Ga-HMFI zeolites exhibited improved selectivity of 75.6% and 78.9%, respectively. Notably, polyaromatic selectivity over Ga-HMFI zeolite declined to 7.3%, significantly lower than that over HZSM-5 (14.9%) and Ga/HZSM-5 (12.5%). These results indicate that the presence of gallium oxides within the micropore channels of Ga-HMFI zeolite enhances shape selectivity for BTX while suppressing the formation of bulky polyaromatic molecules.

#### 3.2.3. Effects of Polyethylene Content on Catalytic Pyrolysis of Cellulose Co-Feeding with Polyethylene

**Figure 5:**
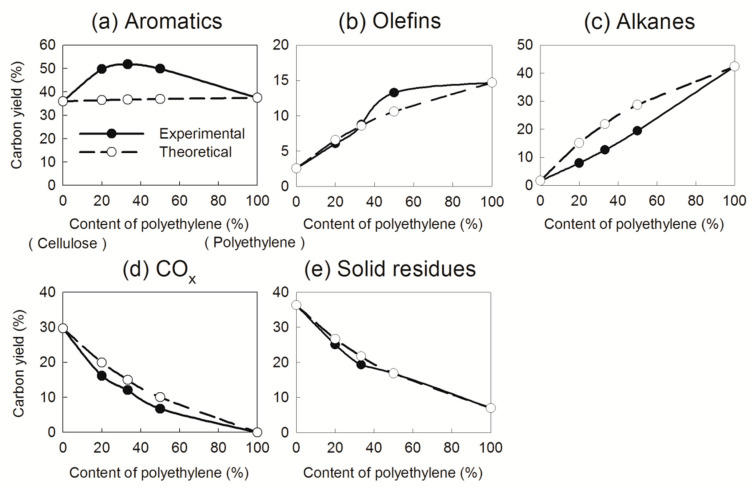
Effects of polyethylene content on product yields in catalytic pyrolysis of cellulose and polyethylene over Ga/HZSM-5 zeolite.

To evaluate the influence of synergy on product distribution during catalytic pyrolysis over Ga/HZSM-5 zeolite, reactants with different mass ratios (4:1, 2:1, and 1:1), corresponding to polyethylene mixing ratios of 20%, 33%, and 50%, were examined (Figure 5). Additionally, the impact of hydrogen content on the interaction between cellulose and polyethylene was assessed by comparing the experimental and theoretical yields of major products. The yields are plotted against the polyethylene content in the mixtures, which increased from 0% to 100%.

If no synergy existed between cellulose and polyethylene, the theoretical and experimental yields would be identical in the catalytic pyrolysis process. However, the experimental yields deviated from the theoretical values, exhibiting a linear correlation with polyethylene content. For aromatics, the experimental yields significantly exceeded the theoretical values (Figure 5a), whereas the experimental yields of alkanes, CO_x_, and solid residues were moderately lower than expected (Figure 5c–e). The yield of petroleum hydrocarbons peaked at 63% when the polyethylene content reached 50%. These trends indicate an interaction between cellulose and polyethylene during catalytic pyrolysis [16], where the increased production of aromatics is likely facilitated by the reduced formation of alkanes, CO/CO_2_, and solid residues.

Previous studies have noted that the interaction between biomass and polyolefin primarily occurs through the Diels–Alder reactions, in which biomass-derived oxygenates react with polyolefin-derived olefins to produce cycloadducts, which subsequently undergo a dehydration reaction to produce aromatics [15,24]. Compared with the individual catalytic pyrolysis of cellulose or polyethylene, the Diels–Alder reaction alters the deoxygenation pathways of oxygenates, shifting the primary mechanism from decarboxylation and decarbonylation to an alternative route during the co-feeding process [16,18]. As a result, more carbon from oxygenates is incorporated into aromatics via the Diels–Alder reaction, rather than being lost as CO_2_ and CO through decarboxylation and decarbonylation in individual catalytic pyrolysis of cellulose (Figure 5d). Furthermore, the Diels–Alder reaction reduces the formation of solid residues that typically result from deoxygenation reactions in the pyrolysis of cellulose alone. Consequently, catalytic pyrolysis of cellulose and polyethylene yields higher amounts of aromatics and fewer solid residues than predicted by theoretical calculations.

**Figure 6:**
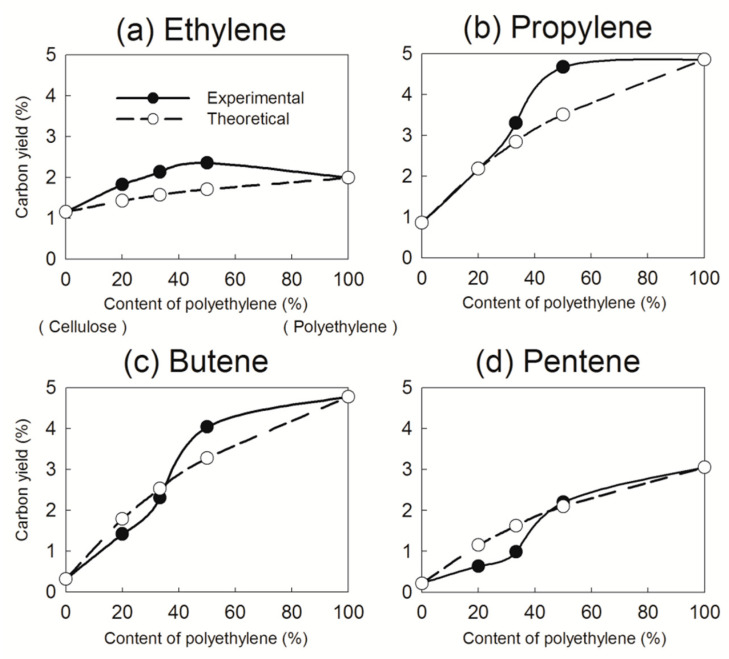
Effects of polyethylene content on olefin yields in catalytic pyrolysis of cellulose and polyethylene over Ga/HZSM-5 zeolite.

For olefins, the experimental yields closely matched the theoretical values at polyethylene contents of 20% and 33% but exceeded them at 50% (Figure 5b). To further examine the role of olefins in the Diels–Alder reaction, the experimental and theoretical yields of major olefins were compared (Figure 6). The experimental yields of butene and pentene were lower than the theoretical values at polyethylene contents of 20% and 33% but exceeded them at 50% (Figure 6c,d). This suggests that olefin yields are highly sensitive to polyethylene content during catalytic pyrolysis. Previous studies [16,25] have indicated that polyethylene-derived butene and pentene undergo conversion with oxygenates to form aromatics at polyethylene contents of 20% and 33% through the Diels–Alder reaction. However, at a polyethylene content of 50%, the excess production of butene and pentene surpasses the reaction capacity of the Diels–Alder pathway, leading to higher experimental yields than theoretical predictions.

Compared with butene and pentene, the experimental yield of ethylene, a lower-carbon olefin, consistently exceeded the theoretical yield (Figure 6a), indicating that ethylene is relatively insensitive to polyethylene content. Because of the absence of alkyl substituents, ethylene does not readily react with oxygenates and is not significantly consumed in the Diels–Alder reaction. These findings align with previous conclusions [33] that catalytic pyrolysis of furans with ethylene does not generate significant synergy for aromatic production, whereas substituting ethylene with propylene substantially enhances aromatic yields. Therefore, optimizing the selection of cellulose and plastic waste is crucial for enhancing synergistic interactions during catalytic pyrolysis.

## 4. Conclusions

The results of this study indicated a significant synergistic effect in the catalytic pyrolysis of cellulose with polyethylene as a hydrogen-rich feedstock. This synergy enhanced interactions between the primary pyrolysis reactants, improving the catalytic conversion of carbon atoms into petrochemicals while suppressing polyaromatic formation. Compared with HZSM-5, both gallium-impregnated Ga/HZSM-5 and hydrothermally synthesized Ga-HMFI zeolites exhibited increased yields of monoaromatics and olefins. These enhancements can be primarily attributed to (i) the narrowed micropores in the zeolites, which inhibited polyaromatic formation and facilitated the diffusion of monoaromatics, and (ii) the presence of gallium oxides, which promoted the dehydrogenation of alkanes to aromatics in catalytic pyrolysis. Therefore, Ga-modified HZSM-5 zeolites are a promising candidate for enhancing the synergy between lignocellulosic biomass and solid waste plastics and optimizing the product distribution toward high-value petrochemicals.

## Figures and Tables

**Table 1:** Characterizations of HZSM-5 and Ga-modified zeolites.

Catalyst	Ga_2_O_3_ (%) ^a^	SiO_2_/(Al_2_O_3_+Ga_2_O_3_)	S_BET_ ^b^ (m^2^/g)	S_micro_ ^c^ (m^2^/g)	V_total_ ^d^ (cm^3^/g)	V_micro_ ^d^ (cm^3^/g)
HZSM-5	-	25.5	355.2	344.4	0.151	0.133
Ga/HZSM-5	4.82	-	349.5	336.3	0.148	0.130
Ga-HMFI	6.08	28.7	350.4	321.3	0.158	0.124

^a^ XRF analysis; ^b^ BET method; ^c^ t-plot method; ^d^ Single-point adsorption method.

**Table 2:** Product yield in individual catalytic pyrolysis of cellulose or polyethylene over HZSM-5 and Ga-modified zeolites.

Yield (C%)	Cellulose			Polyethylene		
	HZSM-5	Ga/HZSM-5	Ga-HMFI	HZSM-5	Ga/HZSM-5	Ga-HMFI
Monoaromatics ^a^	19.2 ± 0.1	25.9 ± 0.1	18.3 ± 0.3	24.2 ± 0.2	34.0 ± 0.4	35.2 ± 0.1
Polyaromatics ^b^	9.7 ± 0.3	10.0 ± 0.1	4.0 ± 0.1	3.1 ± 0.0	3.4 ± 0.1	0.5 ± 0.2
Olefins ^c^	2.7 ± 0.1	2.5 ± 0.1	3.2 ± 0.1	17.7 ± 0.3	14.7 ± 0.6	31.6 ± 0.6
Alkanes ^d^	1.5 ± 0.1	1.6 ± 0.0	0.4 ± 0.0	44.0 ± 0.8	42.4 ± 1.8	25.2 ± 0.1
CO&CO_2_	28.9 ± 0.0	29.7 ± 0.0	28.1 ± 0.2	-	-	-
Solid residues	34.1 ± 1.2	36.3 ± 3.5	30.0 ± 2.1	6.7 ± 0.8	7.0 ± 0.8	2.6 ± 0.4

^a^ Including benzene, toluene, ethylbenzene, xylenes, ethylbenzene, trimethylbenzen, tetramethylbenzenes. ^b^ Including naphthalene, methylnaphthalene, ethylnaphthalene, dimethylnaphthalene, indane, indene. ^c^ Including ethylene, propylene, butene, pentene. ^d^ Including methane, ethane, propane, butane, pentane, and isomers of butane and pentane.

## Data Availability

The data are contained within the article.
